# Study of Diabetic Foot Problems Among Patients Attending the Diabetic Foot Clinic at Royal Hampshire County Hospital, United Kingdom: A Single-Centre Experience

**DOI:** 10.7759/cureus.41454

**Published:** 2023-07-06

**Authors:** Mayurathan Pakkiyaretnam, Jimmy Chong

**Affiliations:** 1 Department of Clinical Sciences, Faculty of Healthcare Sciences, Eastern University Sri Lanka, Batticaloa, LKA; 2 University Medical Unit, Teaching hospital Batticaloa, Batticaloa, LKA; 3 Department of Diabetes and Endocrinology, Royal Hampshire County Hospital, Winchester, GBR

**Keywords:** peripheral arterial disease, peripheral neuropathy, amputation, osteomyelitis, ulcer, diabetes mellitus

## Abstract

Background

Patients with diabetes mellitus (DM) are on the rise all over the world. Simultaneously, the complications of DM are also increasing. Diabetes-related foot problems have been another concern among health professionals, especially foot ulcers, osteomyelitis, and amputations.

Objectives

We determined the prevalence of gender, age, types of DM including non-diabetics, various foot-related presentations, complications, and their outcomes.

Methods

A retrospective descriptive cross-sectional study was conducted among new patients attending a diabetic foot clinic over a period of six months, from January 1, 2019 to June 30, 2019. To confirm the outcome of the study, all of them were followed up for at least four months from the date of diagnosis.

Results

The study showed that most patients were males (65.5%). The most common age group for diabetic foot problems was 81-90 years, and about 80% of the foot problems were diagnosed in patients over 60 years. The study disclosed that 86.2% of the population had type 2 DM, 56.9% had ulcers, and 13.8% had osteomyelitis. The outcome of our study demonstrated that 65.5% of the patients were cured and discharged within four months of the diagnosis, but 10.3% of the population needed amputation. During the four-month follow-up period, 3.4% of our study population died due to non-foot-related causes. A total of 48.1% of our ulcer patients were discharged within eight weeks of diagnosis. However, 26% of ulcer patients and 75% of osteomyelitis patients needed more than four months to be discharged. Peripheral neuropathy and peripheral arterial disease (PAD) were present in 91% of ulcer patients. Among our osteomyelitis group, 100% had peripheral neuropathy, and 87.5% had PAD. About 20% of ulcer patients and none of the osteomyelitis patients were diagnosed with chronic kidney disease (CKD) stages beyond 3b. About 2/3rd of our ulcer and osteomyelitis population had an HbA1C level of more than 7.5%.

Conclusion

Male patients over 60 years of age with type 2 DM are more at risk of developing diabetes-related foot issues. Ulcer with or without osteomyelitis was the most common complication among our study population. Results showed that a significant amount of osteomyelitis patients underwent foot amputation. Poor glycaemic control of HbA1C of more than 7.5%, peripheral neuropathy, and PAD were the most common risk factors for developing foot-related complications. Prolonged use of antibiotics and a dedicated professional team may be needed to manage these complications successfully.

## Introduction

Diabetes mellitus (DM) is a major concern among medical professionals in today's world. Globally, the number of people suffering from DM is rising exponentially. It causes various complications including vision loss, renal failure, peripheral neuropathy, heart attacks, stroke, peripheral vascular disease, and is a major cause of lower limb amputations [1]. Therefore, prevention and early diagnosis are crucial. Foot-related diabetic complications have been well-studied in several aspects. Among diabetic patients, 25% develop foot problems, and about 15% of all hospital admissions among them are directly related to foot infections [2, 3]. Data shows that 1 in 15 DM patients undergo lower limb amputation at some point in their lives, and about half of those with a below-knee amputation end up needing amputation of the other leg within two years. In addition, studies show nearly 100% mortality at five years among diabetic patients with bilateral leg amputations. WHO predicts that by 2030, DM shall be the 7th leading cause of death worldwide [3, 4]. Therefore, good glycemic control is vital in managing patients with DM. Peripheral neuropathy and peripheral arterial disease (PAD) are the fundamental causes of foot problems among diabetic patients [5]. Chronic hyperglycemia in diabetes patients contributes to the accumulation of sorbitol and other metabolically active substances, leading to endo-neuronal hypoxia. They impair the conduction of electrical impulses and facilitate the loss of nerve fibers. This causes peripheral sensory-motor neuropathy and autonomic dysfunction. Autonomic neuropathy impairs a person's ability to sweat and leads to dry skin and the formation of cracks/fissures, which enhances the entry of microorganisms and foot infections [3]. Further, wound healing is affected due to reduced blood supply to the extremities of the lower limbs on account of atherosclerosis-mediated narrowing and microvasculature of the arteries and veins. The pathogenesis involves a complex interplay of multiple factors, including inflammatory markers such as higher levels of IL-6 and resistin and lower levels of adiponectin. They all include chronic uncontrolled hyperglycemia, which has been linked to chronic foot ulcers and the formation of osteomyelitis [3]. Over the last two decades, studies have shown that diabetic foot ulcers shift from neuropathic ulcers to neuro-ischemic ulcers [2, 4]. Surprisingly, inadequate assessment of peripheral neuropathy and PAD, underuse of imaging facilities, and delays in the referral system for revascularization are some of the important reasons for higher amputation rates, in addition to poor glycemic control [5, 6]. Further, osteomyelitis is a major threat contributing to the high risk of amputation. It can be diagnosed clinically and through investigations such as erythrocyte sedimentation rate (ESR), X-ray, CT, or MRI scans [2]. Guidelines usually recommend at least three months of antibiotic treatment for osteomyelitis when surgical treatment is not offered or when infected dead bone residuals remain after surgery. Sometimes a longer duration of therapy, more than three months, may be needed to completely cure osteomyelitis [2, 7]. Hence, appropriate usage of existing facilities is pivotal in preventing and delaying all varieties of diabetic complications, including foot-related problems.

## Materials and methods

The study aims to determine the prevalence of diabetic-related foot problems and associated complications. This was a retrospective, descriptive cross-sectional study conducted among patients attending a diabetic foot clinic at Royal Hampshire County Hospital (RHCH), United Kingdom, over a six-month period, from January 1, 2019 to June 30, 2019. The study period was chosen well before the onset of the COVID-19 infection to recruit adequate samples. The objective of the study was to determine the prevalence of gender, age, and types of DM, including non-diabetic, various foot-related presentations and their outcomes, complications of diabetes such as peripheral neuropathy, PAD, and diabetic nephropathy, and control of DM (by assessing HbA1C levels) among patients attending the diabetic foot clinic. In addition, we analyzed diabetic foot ulcers and osteomyelitis separately, as we noticed these were the most common problems among our clinic attendees at RHCH as per our pilot study. Only the new patients attending the foot clinic were included, and all of them were followed up for at least four months from the date of diagnosis to confirm the study's outcome. It is a multidisciplinary clinic with endocrinologists, podiatrists, and orthopedic teams. The data extraction sheet was prepared according to the specific objectives and data that were retrieved from the medical records of the diabetic foot clinic. We collected data on age, gender, type of DM, and various presentations of foot-related problems from the medical records. These data were analyzed at the end of a four-month follow-up to decide the outcome of the patients. X-ray with or without an MRI scan was used to confirm osteomyelitis in addition to the clinical features. Peripheral neuropathy was assessed by the 10 g monofilament test, and PAD was assessed by checking the patient's pulse using a hand Doppler and CT angiogram (only for selected patients). Diabetic nephropathy was studied according to the estimated glomerular filtration rate (eGFR) and classified into stages 1-5 of chronic kidney disease (CKD) per the international guide. Blood sugar control was analyzed according to patients' HbA1C levels. The outcome of the study was defined as discharged (cured), ongoing treatment (at four months), referred to surgical team for treatment and cured, amputated (toe or foot), or died.

## Results

The total number of new patients during our study period was 58, of which 38 (65.5%) were male and 20 (34.5%) were female. Age distribution showed an equal proportion with one (1.7%) participant each under the age group of 31-40, 41-50, and 91-100 years. Further, the study included 9 (15.6%), 14 (24.1%), and 13 (22.4%) participants under the age groups of 51-60, 61-70, and 71-80 years, respectively. The highest proportion of participants was observed under the age group of 81-90 years, with 19 (32.8%) participants. None of the participants were less than 30 or more than 100 years. Distribution of DM types revealed that three (5.2%) patients had type 1 DM, and the majority, i.e., 50 (86.2%), had type 2 DM. Only one (1.7%) patient had gestational DM. Interestingly, four (6.9%) of the patients were not affected by DM. According to our study, the majority of patients, i.e., 33 (56.9%), presented with foot ulcers (without osteomyelitis) and eight (13.8%) with osteomyelitis. Three patients (5.2%) of the population had Charcot arthropathy, and eight (13.8%) had a callus on their sole. At the time of the clinic visit, two (3.4%) patients in our study group had dry gangrene, while three (5.2%) had symptomatic peripheral neuropathy. Only one (1.7%) patient had acute cellulitis at the presentation time. All the non-diabetic patients had foot ulcers only. Further evaluation demonstrated that 51 (88%) of the new patients had peripheral neuropathy (symptomatic or asymptomatic), and 48 (83%) had PAD. Diabetic nephropathy was studied by analyzing eGFR levels. It indicated that in the total population, 15 (25.9%) patients had an eGFR of above 90 ml/min/m2 (CKD stage 1), 20 (34.5%) had an eGFR level of 60-89 (CKD stage 2), 13 (22.4%) and 5 (8.6%) had an eGFR of 45-59 ml/min/m2 (CKD stage 3a) and 30-44 ml/min/m2 (CKD stage 3b), respectively. Further, only one (1.7%) patient had an eGFR of 15-29 ml/min/m2 (CKD stage 4), while four (6.9%) had an eGFR of less than 15 ml/min/m2 (CKD stage 5). Our patients' sugar control was studied by analyzing their HbA1C levels. It highlighted that five (8.6%) patients from the population had an HbA1C level of less than 5.5%. Fourteen patients from the population (24.1%), which was the highest population, had HbA1C levels between 5.6 and 6.5%. Within the same population, nine (15.6%) had HbA1C levels of 6.6-7.5%, 10 (17.2%) had an HbA1C level of 7.6-8.5%, nine (15.5%) had an HbA1C level of 8.6-9.5%, seven (12.1%) had an HbA1C level of 9.6-10.5%, and one (1.7%) had an HbA1C level of 10.6-11.5%, while three (5.2%) had an HbA1C level of 11.6-12.5%. None of them had an HbA1C level of more than 12.6%. Our study's outcome illustrated that most patients (38; 65.5%) were cured and discharged. At four months from the time of the diagnosis, eight (13.8%) patients had ongoing treatment. Additionally, four (6.9%) of our patients were referred to the surgical team for treatment and discharged from the clinic after the cure. Unfortunately, six (10.3%) patients needed amputation of the toe or foot during our study period of four months, and two (3.4%) patients from our study group died within this study period. Further inquiry revealed that the cause of death was not directly connected to their foot problems.

Foot ulcers (33; 56.9%) and osteomyelitis (8; 13.8%) were the most common presentations among our subjects. Therefore, we analyzed both of these conditions separately. Most ulcer patients were male (24; 72.2%), and most females were found in the osteomyelitis group (5; 62.5%). As indicated, a significant number of male patients presented with ulcers (df = 1, p-value = 0.00). The most common age group for ulcer patients was 61-70 years (10; 30.3%) and osteomyelitis patients were 81-90 years (5; 62.5%). Both were common among type 2 DM patients, with 30 (91%) and 7 (87.5%) patients scoring ulcer and osteomyelitis, respectively (Table 1).

**Table 1:** Prevalence of osteomyelitis and ulcer according to gender, age, and type of DM. DM: Diabetes mellitus.

Characteristics	Osteomyelitis	Ulcer
Gender		
Male	3 (37.5%)	24 (72.2%)
Female	5 (62.5%)	9 (27.8%)
Age		
31-40 years	0	1 (3.0%)
41-50 years	0	0
51-60 years	1 (12.5%)	6 (18.2%)
61-70 years	2 (25.0%)	10 (30.3%)
71-80 years	0	6 (18.2%)
81-90 years	5 (62.5%)	9 (27.3%)
91-100 years	0	1 (3.0%)
Type of DM		
Type 1 DM	1 (12.0%)	1 (3.0%)
Type 2 DM	7 (87.5%)	30 (91.0%)
Gestational DM	0	1 (3.0%)
No DM	0	1 (3.0%)

The outcome of our study showed that 20 (60.6%) of the patients with ulcers and four (50%) with osteomyelitis were discharged at the time of analysis. However, four (12.1%) other patients with ulcers and three (37.5%) with osteomyelitis needed surgical amputation. Further, it showed that a significant number of amputations occurred among the osteomyelitis group compared to the ulcer group (df = 4, p-value = 0.00). According to the data, one (3%) patient with an ulcer died (due to other causes), and no death was reported among the osteomyelitis group (Table 2).

**Table 2:** Outcome of osteomyelitis and ulcer patients.

Outcome	Osteomyelitis	Ulcer
Discharged	4 (50.0%)	20 (60.6%)
Ongoing treatment at four months	1 (12.5%)	7 (21.2%)
Amputated	3 (37.5%)	4 (12.1%)
Surgical management without amputation	0	1 (3.0%)
Died	0	1 (3.0%)

**Figure 1:**
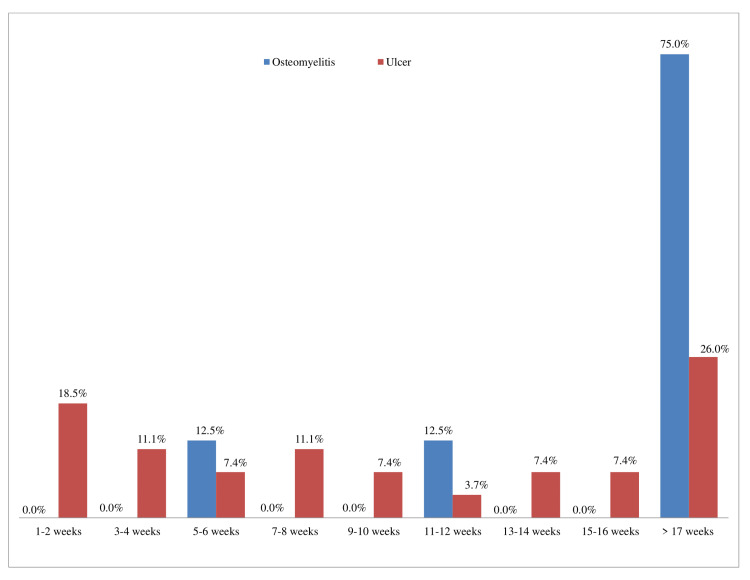
Time duration of patients’ discharge.

The time duration to discharge the patients is demonstrated in the bar chart (Figure 1) below. The data revealed that most osteomyelitis (75%) and ulcer (26%) patients needed more than four months to heal and get discharged from the clinic.

**Figure 2:**
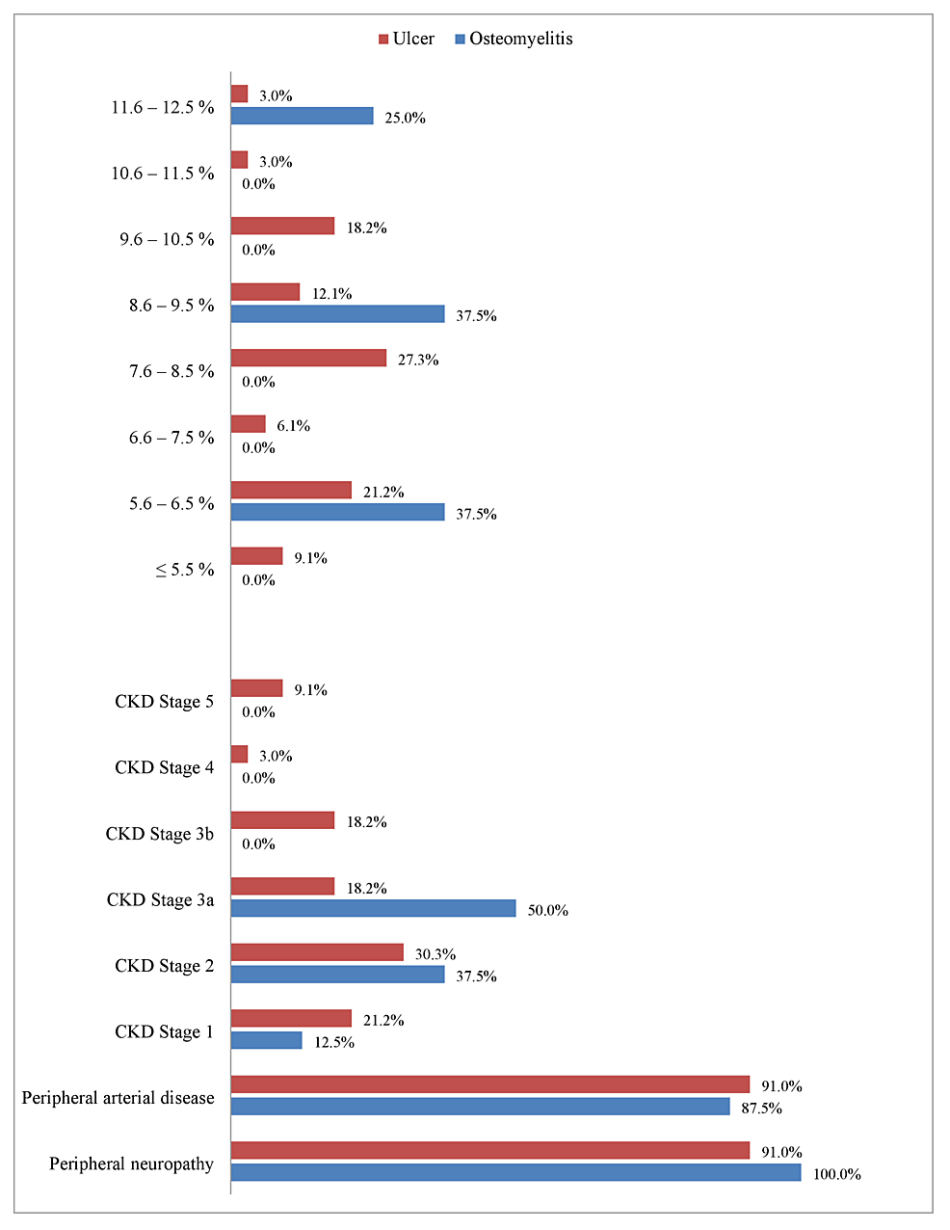
Comparison of HbA1C levels (in percentages), CKD stages, peripheral neuropathy, and peripheral arterial disease between patients with ulcers and osteomyelitis. CKD: Chronic kidney disease.

Complications of DM such as peripheral neuropathy, PAD, and diabetic nephropathy among studied patients population with ulcer and osteomyelitis and their HbA1C levels are demonstrated in Figure 2. Most ulcer patients (9; 27.3%) had HbA1C levels between 7.6 and 8.5%, while most osteomyelitis patients (3; 37.5%) had HbA1C levels between 5.6-6.5% and 8.6-9.5%. Furthermore, 30 (91%) ulcer patients and all osteomyelitis patients had peripheral neuropathy. Among these, all ulcer patients (30; 91%) and seven (87.5%) osteomyelitis patients were affected by PAD. The majority of ulcer patients (10; 30.3%) fell below the stage 2 CKD level, and four (50%) osteomyelitis patients were under stage 3a CKD level. Interestingly, only four (20%) ulcer patients and none of the osteomyelitis patients had a CKD level beyond 3b.

## Discussion

Our study showed that most patients (65.5%) were males. Similar data from other studies indicated that 2/3rd of diabetic foot issues occurred among males [8, 9, 10, 11]. However, one study conducted in Pakistan on the risk factors for diabetic foot ulcers showed that 80.1% of the patients were male [12]. According to our study, the most common age group for diabetic foot problems was 81-90 years, and about 80% of the foot problems occurred among patients over the age of 60 years. A study by Wasim Ahmad et al. done at Ayub Medical College Hospital, Pakistan, revealed that 74.4% of the patients in the study were between 40 and 70 years [12]. Another study by Milne TE et al. conducted at the Royal Perth Hospital's Podiatry Department in Western Australia, showed that a mean age of 62 +/- 12 years was common in the patients who developed diabetic foot ulcers [10]. Therefore, it is true that men over 60 years with DM are at high risk of developing foot problems [8]. Almost all the studies in the literature were conducted among type 2 diabetes patients. Our study disclosed that 86.2% of the population had type 2 DM. A study in Karachi states that 99% of foot ulcers occurred in patients with type 2 DM [9]. In addition, 1.7% of the patients had gestational DM, and interestingly, 6.9% had no DM, according to our study. Unfortunately, there were not many studies in the literature about various foot presentations of DM. Almost all the studies evaluated foot ulcers and osteomyelitis as common presentations [13, 14, 15]. Similarly, our study also depicted that these conditions were more than 70%, with ulcer accounting for 56.9% and osteomyelitis for 13.8%. It is evident that ulcers are the most common manifestation of a diabetic foot compared to osteomyelitis, even though both are more common than all other conditions. It is estimated that osteomyelitis in DM is about 15% [8]. Our study also supports this finding. The outcome of our study demonstrated that 65.5% of patients were cured and discharged within four months of diagnosis. It is estimated that about 25% of diabetes patients need amputation of their legs [8]. A large cohort prospective study with 2138 samples conducted in Jabir Abu Eliz Diabetic Center, Khartoum, Sudan, on diabetic foot infections with osteomyelitis revealed that 15.8% of the total patients required amputation [14]. However, in our study, only 10.3% of the population needed amputation. Due to non-foot-related causes, 3.4% of our study population died during the four months of the follow-up period. An almost similar death rate among diabetic ulcer patients was observed in a summary report on the quality of foot care for people with diabetes, based on findings of the National Diabetes Foot Care Audit (NDFA) 2014-16 in England and Wales. The report showed 304 deaths among 13,034 ulcer cases (2.33%) within 12 weeks of follow-up [15].

As per the literature, the usual healing time for an ulcer is typically a few weeks, while that for osteomyelitis is around three months or more [2, 7]. Around 48.1% of our ulcer patients were discharged within eight weeks of diagnosis. However, a surprisingly significant number of ulcer patients (26%) and most osteomyelitis patients (75%) needed more than four months to get discharged. To fully comprehend the reasons for the long duration of treatment, further studies need to be conducted. Interestingly, a similar long-term treatment was found to be necessary in a UK study with 154 patients. A study conducted by Ince P et al. showed that ulcer healing was 59.3%, 70.5%, and 86.6% at 12, 20, and 52 weeks, respectively [11]. In previous studies, the prevalence of peripheral neuropathy was about 42-51%, while that of PAD was about 58-63% [9, 12]. However, in our osteomyelitis group, 91% of ulcer patients had peripheral neuropathy and PAD, 100% had peripheral neuropathy, and 87.5% had PAD. Again, further studies are needed to identify the reasons and immediate action plans to overcome the issues. Interestingly, only about 20% of ulcer patients and none of the osteomyelitis patients had CKD levels beyond 3b. Another important finding was that about 2/3rd of our ulcer and osteomyelitis population had an HbA1C level of more than 7.5%. This indicates that poor glycemic control is an important risk factor for ulcers and osteomyelitis [5, 9].

## Conclusions

Diabetes-related foot problems are more prevalent among men over 60 years of age with type 2 DM. The most common complication among patients attending our foot clinic was an ulcer, with or without osteomyelitis. Amputation emerged as a serious outcome for a significant number of patients with osteomyelitis. Poor glycemic control (HbA1C levels of more than 7.5%), peripheral neuropathy, and PAD are the most common risk factors for developing foot-related complications. A long duration of antibiotics and a dedicated professional team may be needed to manage these complications successfully. We recommend that a similar study be conducted in the future with a larger sample size to further elaborate on the outcomes.
